# Global Trends in Tai Chi Research: A Bibliometric Analysis

**DOI:** 10.3390/sports14010014

**Published:** 2026-01-04

**Authors:** Tzu-Yu Huang, Wei-Li Hsieh, Kai-Yuan Cheng, Marius Brazaitis, Chen-Sin Hung, Ruei-Hong Li, Shih-Chun Kao, Ngoc Thi Bich Tran, Yu-Kai Chang

**Affiliations:** 1 Department of Physical Education and Sports Sciences, National Taiwan Normal University, Taipei 106, Taiwan; tyhuang86@gmail.com (T.-Y.H.); lily60380@gmail.com (W.-L.H.); chensinhung.0211@gmail.com (C.-S.H.); rueihongli@gmail.com (R.-H.L.); 2 Office of Physical Education, National Defense University, Taoyuan 334, Taiwan; 3 Institute of Philosophy of Mind and Cognition, National Yang Ming Chiao Tung University, Hsinchu 300, Taiwan; kcheng1970@nycu.edu.tw; 4 Sports Science and Innovation Institute, Lithuanian Sports University, LT-44221 Kaunas, Lithuania; marius.brazaitis@lsu.lt; 5 Department of Health and Kinesiology, Purdue University, West Lafayette, IN 47907, USA; kao28@purdue.edu; 6 Institute of Social Sciences, Hanoi National University of Education, Hanoi 100000, Vietnam; ngocttb@hnue.edu.vn; 7 Faculty of Education Management, Hanoi National University of Education, Hanoi 100000, Vietnam; 8 Social Emotional Education and Development Center, National Taiwan Normal University, Taipei 106, Taiwan

**Keywords:** visualization, Taichi, Traditional Chinese exercises, mind–body exercise, complementary medicine

## Abstract

Tai Chi has evolved into a widely used mind–body practice increasingly incorporated into complementary therapy, rehabilitation, and public health. This study provides an updated global bibliometric overview, with VOSviewer mapping publication performance, co-authorship networks, and keyword-based thematic clusters. Articles and reviews with Tai Chi–related terms in the title were retrieved from Scopus, with no restrictions on language or publication year. A total of 2253 publications from 1978 to 2025 were analyzed, revealing steady growth, concentrated largely in the past decade. China led the publication output, while the United States had the highest number of citations, forming a dual-core pattern. The field is largely driven by a small group of authors and regional clusters, and its visibility in mainstream medical journals remains limited. Nine software-generated keyword clusters were manually synthesized into five themes: motor function (balance and fall prevention), musculoskeletal conditions (osteoarthritis, rheumatoid arthritis, fibromyalgia), chronic disease management (cardiovascular disease, stroke, COPD), psychological health (quality of life, depression, anxiety, mindfulness), and cognitive aging (dementia, mild cognitive impairment). Future progress requires greater methodological rigor, including mechanistic inquiry, long-term study designs, and community- or population-level applications, along with stronger international collaboration and deeper integration into clinical and public health practice.

## 1. Introduction

Increasing population-level physical activity is now recognized as a core priority in public health policy. Regular physical activity is widely regarded as an effective, safe, low-cost, and sustainable non-pharmacological strategy to prevent chronic diseases, maintain physical function, and enhance overall quality of life [1]. However, participation in physical activity or structured exercise programs is often limited by barriers related to accessibility, physical capability, motivation, and practical constraints [2]. In this context, movement-based mind–body therapies, such as yoga, qigong, and Tai Chi, have gained increasing attention owing to their gentle intensity, adaptability, and suitability for individuals who have difficulty engaging in conventional exercise. Tai Chi (also known as “Tai Chi Chuan” or “Taijiquan”), with its low-impact movements, minimal equipment requirements, and self-paced nature, is a notable example of this trend.

Tai Chi, blending philosophy, martial arts, and health cultivation, offers unique values that set it apart from traditional exercise modalities [3]. Its frequent weight shifting, single-leg stances, and controlled postures effectively strengthen the lower limbs and core stability, thereby improving the posture and balance [4]. Characterized by slow and neuromuscular coordination, breathing, and mindful intention guiding, Tai Chi embodies a form of mind–body integration [5]. Beyond physical benefits, regular Tai Chi practice reduces stress, promotes emotional regulation, and supports cognitive functions, such as attention and memory [6], reinforcing its role as a comprehensive mind–body exercise with broad applicability across health and rehabilitative contexts.

Tai Chi has drawn increasing scholarly attention across medicine, public health, exercise science, and the humanities. While this diversity of disciplinary perspectives underscores the richness of Tai Chi research [7,8,9,10], it has also produced a fragmented and heterogeneous body of knowledge that is difficult to synthesize. To address this challenge, bibliometric analysis offers a systematic and quantitative means of integrating diverse research strands, enabling a comprehensive understanding of publication patterns, knowledge structures and thematic trajectories. This approach has proven valuable for mapping research landscapes and informing future agendas in scientific disciplines [11,12]. Several bibliometric studies have examined Tai Chi in relation to specific health domains, such as cognitive function [13], fall prevention [14], neurodegenerative diseases [15], osteoporosis [16] and insomnia [17], while a few have attempted to provide broader, field-level overviews of Tai Chi research itself [18,19,20,21,22] (see Table 1). As summarized in Table 1, however, these analyses are constrained by outdated [18,19,20,21] and relatively short coverage periods [19,22], and narrow Tai Chi-related keyword strategies [22]. These limitations underscore the need for an updated, field-wide bibliometric analysis to delineate the field’s overall structure and evolution.

To address these gaps, the present study provides an updated field-wide bibliometric analysis of Tai Chi research. Specifically, we pursue three aims. First, we delineated the field’s growth trajectory and leadership structure across regions and collaboration networks. Second, we examine cross-disciplinary visibility by comparing publication venues and citation patterns across journal categories. Third, we mapped the thematic landscape and its evolution through keyword co-occurrence to identify major domains, emerging fronts, and persistent gaps. By integrating fragmented literature across disciplines, this study clarifies the field’s structural patterns and emerging directions and offers a foundation for future scientific and applied progress in the field.

## 2. Methods

### 2.1. Data Source and Retrieval

An extensive literature search was conducted using the Scopus database, which was chosen for its comprehensive coverage of high-quality peer-reviewed journals across multiple disciplines, including medicine, neuroscience, nursing, health professions, psychology, environmental science, computer science and decision sciences. The database also provides detailed citation indexing, making it particularly suitable for bibliometric analysis [23,24,25,26].

The search and screening procedures followed the Preferred Reporting Items for Systematic Reviews and Meta-Analyses (PRISMA) framework. Keywords used in the search included “Tai Chi,” “Taichi,” “Tai-Chi,” “Tai Ji,” “Taiji,” “Tai-Ji,” “T’ai-Chi,” “Taichiquan,” “Taijiquan,” “Taichichuan,” and “Taijichuan,” which were applied within the Article Title field. These variants capture the common Tai Chi spellings [27,28], ensuring comprehensive retrieval. Restricting the search to the title field minimized thematic dilution and ensured that keyword analysis reflected Tai Chi research. Only documents classified as Articles or Review Articles were included, as they provide the necessary methodological and bibliometric information, whereas conference papers, letters, and editorials often lack sufficient detail. No language restrictions were applied. The search, which was completed on 21 October 2025, imposed no lower limit on the publication year to capture the entire historical trajectory of Tai Chi research. To ensure relevance, two authors (TYH and CSH) independently screened all titles and excluded records where “Tai Chi” referred to non-exercise or non-martial art contexts. Ambiguities were resolved through discussion and consensus with the corresponding author (YKC).

### 2.2. Bibliometric Methods

Publication records, including information on titles, abstracts, journals, authors, keywords, and citations, were retrieved from Scopus. The records were exported in “. csv” format, and organized in Microsoft Excel for preliminary processing, including data cleaning and format adjustments. Bibliometric analyses, comprising performance analysis and science mapping, were conducted using VOSviewer (v1.6.20).

VOSviewer was used to assess publication and citation performance at the country, author, and journal (source) levels, and to visualize co-authorship networks across countries and authors. Author-level bibliographic coupling was used to map groups of researchers who shared similar reference bases. Author identification and disambiguation relied on the Scopus Author ID system. Author keywords were examined for keyword analysis. VOSviewer generates frequency counts and co-occurrence maps, a visualization technique that groups related terms into clusters. The resulting keyword clusters were manually reviewed and consolidated into broader themes, which were assigned descriptive labels to represent the underlying knowledge structures and research trends clearly.

In the resulting network graphs, the node size reflects the number of publications or the frequency of occurrence, and the connecting lines indicate collaboration or reference relationships. Temporal patterns were further examined using the overlay visualization function, in which VOSviewer assigns colors according to the average publication year associated with each item. The color gradient represents the publication timing, ranging from purple/blue in earlier years to yellow in recent years. These tools provide complementary perspectives that enable comprehensive mapping of the structure, dynamics, and emerging frontiers of Tai Chi research.

## 3. Results

### 3.1. Global Publication Trend

**Figure 1:**
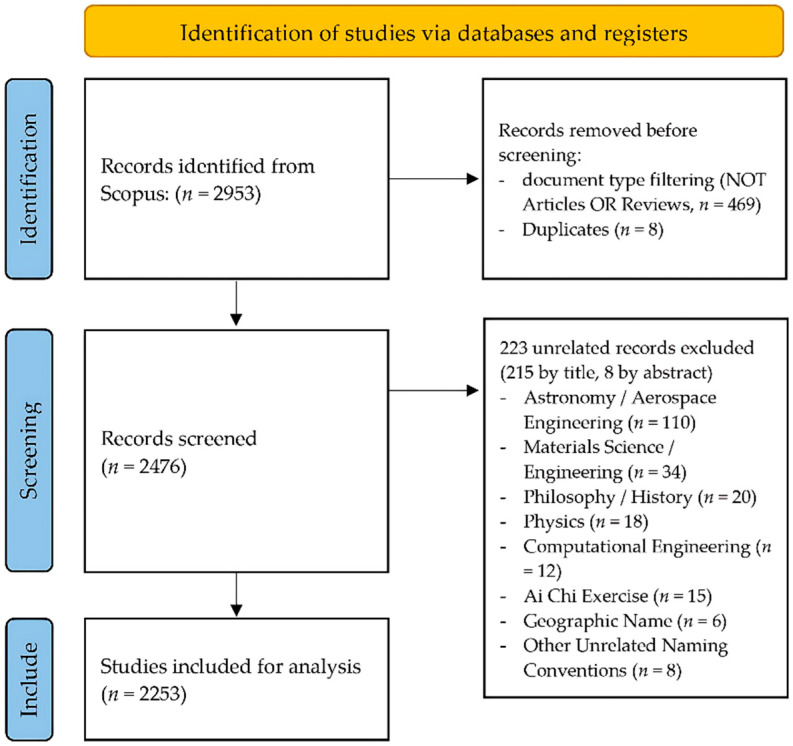
PRISMA flow diagram of the study selection.

A total of 2953 records were retrieved using a keyword search. After filtering for document type, 2086 original research articles and 398 review articles were included for further processing (*n* = 2484). Duplicates (*n* = 8) were removed, and the remaining records were manually reviewed to exclude entries using the terms “Taiji” or “Tai Chi” in non-relevant contexts, including astronomy (e.g., LISA-Taiji), physics, materials science, computer science, philosophy, geographic locations (e.g., Taiji, Japan), and water-based adaptations (e.g., Ai Chi). In total, 223 unrelated records were excluded, leaving a final sample of 2253 publications (1863 articles and 390 reviews) for bibliometric analysis. The two independent screeners reported no discrepancies in the pre-defined criteria. The detailed process is illustrated in Figure 1.

**Figure 2:**
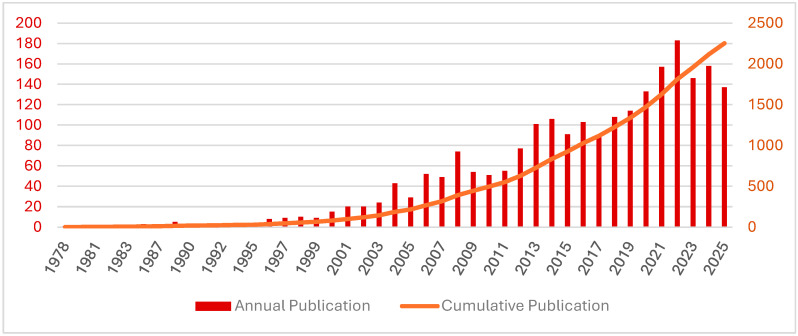
Annual number of publications (articles and reviews) on Tai Chi research.

As identified in this analysis, the literature spans 1978 to 2025 and can be broadly divided into three developmental phases. In the initial phase (1978–1999), the annual publication output remained in single digits. The development phase (2000–2012) was marked by a steeper expansion (compound annual growth rate, CAGR ≈ 15%), with annual counts exceeding 20 by the early 2000s, reaching a temporary peak of 77 publications in 2012, with an average of approximately 43 publications per year. From 2013 onward, the field entered a high-volume phase in which annual output consistently exceeded 100 publications. Between 2013 and 2024, the number of papers increased from 101 to 158 (CAGR ≈ 4%), reaching a peak of 183 publications in 2022 and subsequently fluctuating at a similarly high level. More than 70% of all Tai Chi publications were published during this period. By October 2025, the cumulative number of publications reached 2253 and the average annual output during the study period was approximately 52 (Figure 2).

### 3.2. Analysis of Country and Region

**Figure 3:**
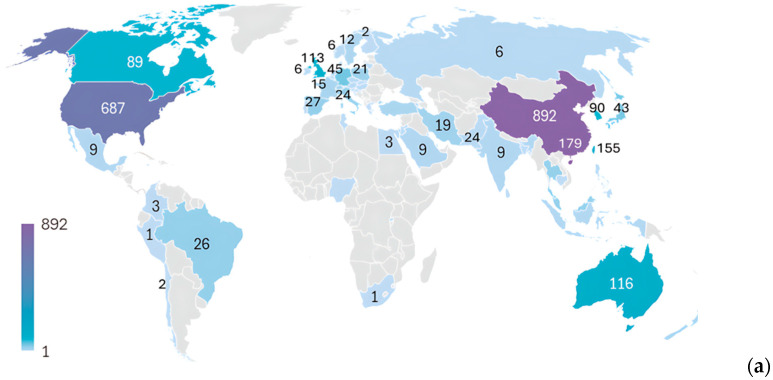

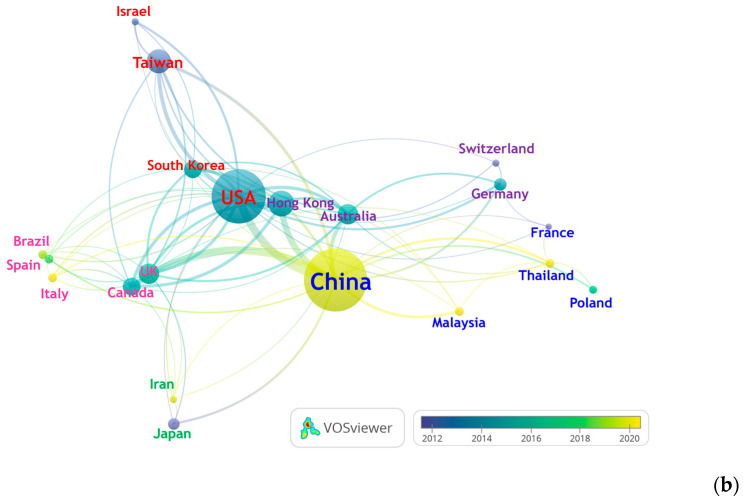
(**a**) Worldwide publication output in Tai Chi research. The numbers indicate the cumulative number of publications by country/region. (**b**) International collaboration networks illustrating clusters (word colors, *n* = 5) and temporal trends (color gradient, 2012–2020).

A total of 76 countries/regions contributed to the publications on Tai Chi. The top 15 countries, based on publication output are listed in Table 2. China ranked first with 892 articles (40%), followed by the United States with 687 (30%). Hong Kong (179), Taiwan (155), Australia (116), and the United Kingdom (113) ranked third to sixth, respectively. Other Asian countries, such as South Korea (90), Japan (43), Thailand (26), and Malaysia (26), have maintained steady output levels. American and European countries, including Canada (89), Brazil (26), Germany (45), Spain (27), and Italy (24), also participated actively (Figure 3). In terms of total citations, the United States stood out with 29,485, surpassing China’s 15,841, despite its higher publication count. Hong Kong and Taiwan accumulated 7764 and 5335 citations, respectively.

To capture the patterns of international engagement further, a country co-authorship network was constructed with a minimum of 15 publications, yielding 20 contributing countries (Figure 3). The results indicate that the United States and China were the two central hubs of international collaboration. The United States clustered with Taiwan, South Korea, and Israel (red cluster), whereas China grouped with Malaysia, Thailand, France, and Poland (blue cluster). Another cluster linked Hong Kong, Australia, Germany, and Switzerland (purple cluster). In addition, a transatlantic collaboration was observed, bringing together the United Kingdom, Canada, Brazil, Spain, and Italy (pink cluster). Japan and Iran have formed smaller subnetwork (green cluster). The temporal overlay shows that collaborations involving China were more recent, occurring closer to 2020, whereas those linked to the United States, Taiwan, Japan, Israel, and France emerged earlier (around 2012–2014).

### 3.3. Analysis of the Authors

Across all included publications, 6977 authors contributed to the field. Peter M. Wayne ranked first in both publication volume and citation count, with 78 articles and 4032 citations. Fuzhong Li (35 articles, 3176 citations) and Peter A. Harmer (26 articles, 2891 citations) also demonstrated both productivity and influence. Gloria Y. Yeh (40 articles, 2210 citations) and William W. N. Tsang (36 articles, 1404 citations) held the second and third positions in publication output, respectively. Steven L. Wolf (21 articles, 3189 citations) and Elizabeth N. Eckstrom (10 articles, 1535 citations) stood out for exceptionally high citation impact, each averaging over 150 citations per publication. Other notable contributors included Chenchen Wang (25 articles, 2323 citations), Ruth E. Taylor-Piliae (30 articles, 1606 citations), and Ching Lan (23 articles, 1863 citations).

**Figure 4:**
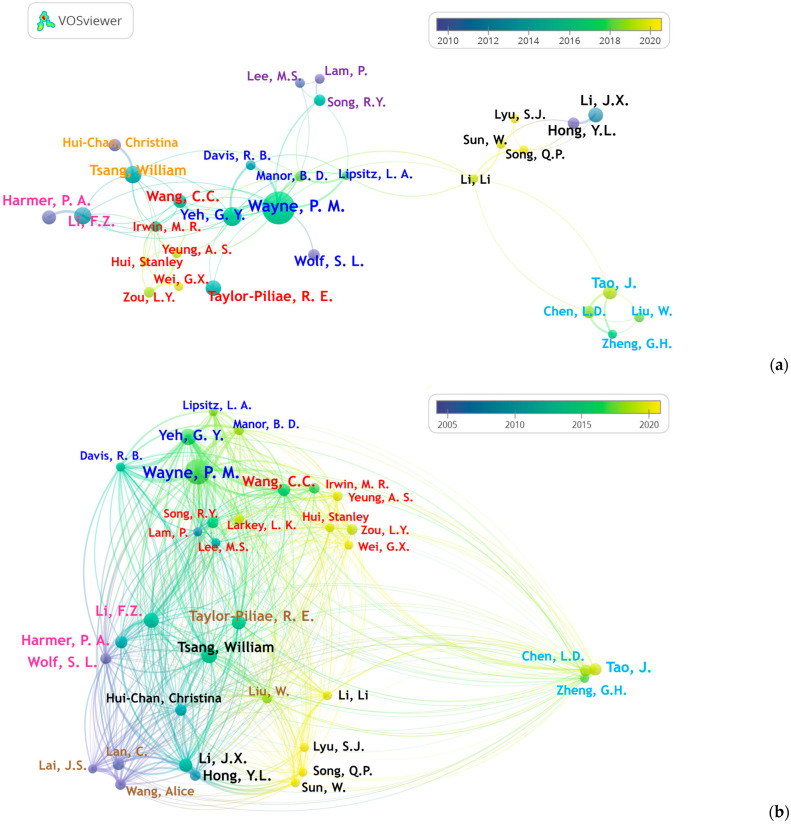
(**a**) Author collaboration network showing clusters (node colors, *n* = 7) and temporal trends (color gradient, 2010–2020); (**b**) author bibliographic coupling network showing clusters (node colors, *n* = 6) and temporal trends (color gradient, 2005–2020).

To examine collaboration patterns, a minimum threshold of 15 publications was applied, yielding 34 authors, of whom 30 were connected to the most extensive collaboration network (Figure 4). The co-authorship analysis identified seven distinct clusters with high regional concentrations. The central blue cluster, led by Peter M. Wayne and Gloria Y. Yeh, represented the largest group. Bridging authors, such as Chenchen Wang (red) and Michael R. Irwin (red), Fuzhong Li (pink), William Tsang (orange; black), and Li Li (black) linked otherwise separate groups. The black and aqua clusters reflect the strong collaboration among Chinese institutions. A smaller purple cluster, centered on Myeong Soo Lee, Rhayun Song, and Paul Lam, represented another active research group in Asia. Bibliographic coupling analysis produced a similar cluster structure (Figure 4b) with six clusters. The temporal overlay further indicates that earlier collaborations were concentrated in North America, such as those involving Peter A. Harmer and Steven L. Wolf. In contrast, more recent partnerships have emerged among Chinese researchers.

### 3.4. Analysis of Documents

Among the 2253 documents analyzed, citation counts ranged from 0 to 963, with a total of 62,055 citations and an average of 27.5 citations per article. A total of 277 papers were uncited. The top 10% of documents received an average of 140 citations, including 131 papers cited over 100 times and 11 papers cited over 300 times (Table 3). Collectively, these publications yielded an h-index of 110. The top three most-cited studies are as follows: Wolf, Barnhart [29] (963 citations), one of the first rigorous randomized trials in this field, demonstrated that a 15-week Tai Chi program reduced recurrent falls while also improving frailty-related and psychosocial outcomes, thereby establishing Tai Chi’s potential as a scientifically validated intervention; Li, Harmer [30] (676 citations), which found that Tai Chi improved postural stability, gait, and functional capacity in patients with Parkinson’s disease while reducing falls; and Li et al. [31] (556 citations), which reported that a six-month Tai Chi program lowered fall incidence and enhanced balance in high-risk community-dwelling elders. In addition, four review articles ranked among the top ten most-cited papers, addressing the comprehensive health benefits of Tai Chi [32] and its effects on chronic conditions [33], psychological well-being [34], and cognitive performance [35].

### 3.5. Analysis of Journals

A total of 842 journals contributed to the publications in this field. Of these, 300 journals published at least two papers, 87 published five or more, and 25 journals produced 15 or more articles. The top 15 journals accounted for 22% of total publications (Table 4), with *Evidence-Based Complementary and Alternative Medicine* ranking first in output (64 articles), followed by *the Journal of Alternative and Complementary Medicine*, *Complementary Therapies in Medicine*, *Medicine*, and *the International Journal of Environmental Research and Public Health*. In terms of citation counts, *the Journal of the American Geriatrics Society had the highest number of total citations* (24 articles, 4437 citations), whereas *the Archives of Physical Medicine and Rehabilitation*, *Journal of Alternative and Complementary Medicine*, and *Evidence-Based Complementary Medicine* also received substantial attention. Notably, although *Medicine and Science in Sports and Exercise* (10 articles, 1203 citations) and *the British Journal of Sports Medicine* (12 articles, 1244 citations) published relatively few papers, they exhibited outstanding performance in terms of average citations per paper.

### 3.6. Analysis of Keywords

**Figure 5:**
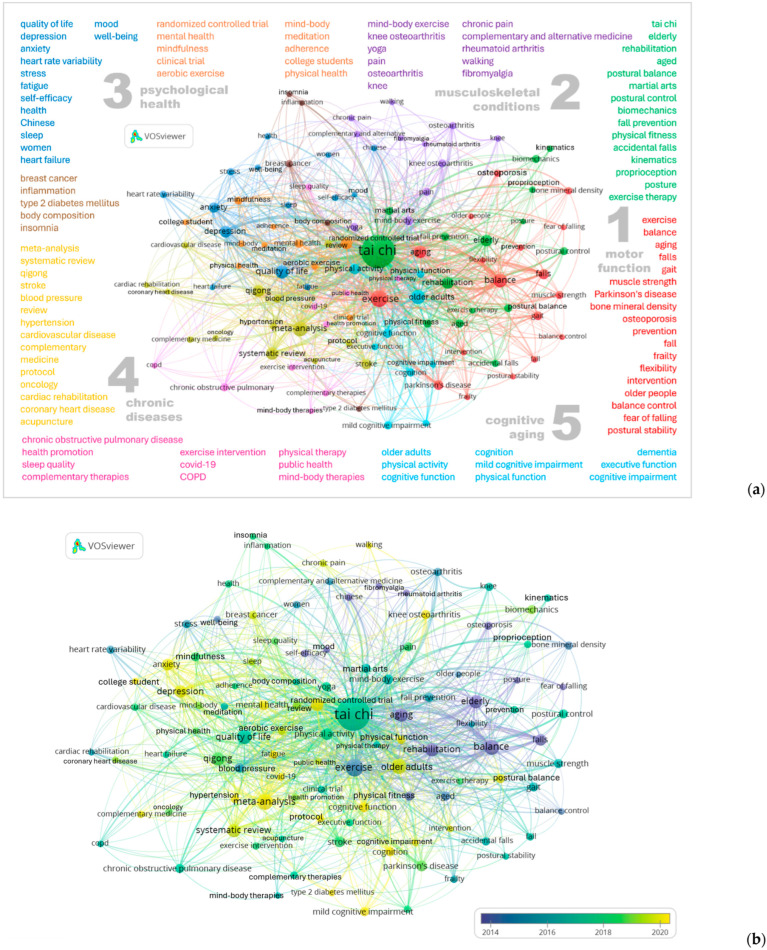
(**a**) Author keyword co-occurrence network in Tai Chi research (*n* = 106). The network displayed five themes derived from nine clusters. Keywords within each cluster are arranged from top to bottom and left to right according to their frequency of occurrence. (**b**) Temporal presentation of the author keywords co-occurrence network.

To maintain an analytical focus, this study used author keywords as the primary source of keyword analysis. A total of 3032 author keywords were identified in this study. After applying term unification to consolidate Tai Chi–related expressions, a minimum occurrence threshold of 10 was set, resulting in the inclusion of 106 keywords for analysis. Excluding Tai Chi itself, the top ten author keywords were *older adults/aging/elderly*, *exercise*, *meta-analysis*, *balance*, *quality of life*, *systematic review*, *qigong*, *rehabilitation*, *randomized controlled trial*, and *depression*. The co-occurrence network revealed nine distinct clusters, which were subsequently summarized into five overarching themes to facilitate interpretation: motor function, musculoskeletal conditions, psychological health, chronic diseases, and cognitive aging (Figure 5).

At the center of Figure 5, *Tai Chi* appears as the most frequently co-occurring term, forming a green cluster with *elderly*, *rehabilitation*, *postural balance*, and *martial arts*. Closely connected to it, the red cluster included *falls*, *gait*, *muscle strength*, *Parkinson’s disease*, and *bone mineral density*. Together, these two clusters represent the “motor function” domain, highlighting the functional benefits of Tai Chi in older adults. In addition, the purple cluster featured “musculoskeletal conditions” such as *knee osteoarthritis*, *rheumatoid arthritis, and fibromyalgia*, which appeared alongside *mind–body exercise* and *yoga*. Themes related to “psychological health,” such as *quality of life*, *depression*, *anxiety*, *stress*, *fatigue*, *self-efficacy*, and *well-being*, constituted the blue cluster, while the orange cluster encompassed *randomized controlled trial*, *mental health*, *mindfulness*, and *college student*. The brown, pink, and yellow clusters covered a range of “chronic diseases,” including *breast cancer*, *type 2 diabetes mellitus*, *chronic obstructive pulmonary disease*, *stroke*, *hypertension*, *cardiovascular disease*, *oncology*, and *COVID-19*. The yellow cluster also featured methodological terms such as *meta-analysis* and *systematic review*. Finally, the aqua cluster focused on “cognitive aging,” comprising *cognition*, *mild cognitive impairment*, *dementia*, and *executive function*.

In addition to structural clustering, an overlay visualization was generated to display the temporal distribution of keywords, where colors ranged from purple (earlier) to yellow (more recent) according to the average publication year (Figure 5). Keywords such as *rehabilitation*, *balance*, *fall*, *elderly*, *osteoporosis*, *posture*, and *self-efficacy* were mapped in purple, representing earlier studies. Terms including *blood pressure*, *stress*, and *heart rate variability* appeared in blue, while *quality of life*, *stroke*, and *Parkinson’s disease* appeared in green. The most recent terms, displayed in yellow, were *meta-analysis*, *systematic review*, *depression*, and *cognitive function*.

## 4. Discussion

In this bibliometric analysis, 2253 publications were analyzed to illustrate the global evolution of Tai Chi research. Since the late 1970s, this field has expanded steadily, with most publications produced in the past decade. The temporal pattern broadly parallels the findings reported by Zhang [40] for wider mind–body therapy literature, which also showed accelerated growth during the 2010s and a local peak in the early 2020s.

### 4.1. Global Patterns and Research Leadership

At the national level, China dominates output, while the United States achieves higher citation impact, indicating greater global visibility, which may partly be attributable to the pioneering role of U.S.-based teams in study scale and methodological rigor [21]. Hong Kong and Taiwan ranked third and fourth in both publication output and citation count, respectively, and together with mainland China, they accounted for more than half of the global publications. This configuration not only underscores their academic prominence, but also highlights the enduring influence of Tai Chi’s deep cultural embeddedness in East Asia. Consistent with previous studies [20,21,22], collaboration networks indicate that China and the United States are the dominant hubs, whereas trans-Pacific and European countries are in secondary clusters, with influence primarily mediated through their ties to these two centers.

At the author level, although nearly 7000 scholars have contributed to Tai Chi research, the field’s development has been driven largely by a relatively small group of influential researchers, including Wayne and Yeh, Tsang, Wang, and Li. Bibliographic coupling revealed cluster structures that closely mirrored those observed in the co-authorship map, indicating that collaborative teams in Tai Chi research also function as cohesive intellectual communities built on highly overlapping reference bases. These patterns suggest that the field is organized around a limited number of well-established, thematically focused research clusters.

The evolution of Tai Chi research, as reflected in country- and author-level temporal trends, is evident in shifting regional contributions. The United States spearheaded the early exploration of this field, paving the way for the gradual expansion of Europe. Recently, China has established new research networks. Overall, Chinese and U.S.-based teams share a convergent intellectual base and have formed mature, internally cohesive communities. While this supports sustained subfield programs, it may also reinforce regional silos, slowing cross-regional synthesis and independent replication. As more countries engage in Tai Chi research, strengthening interregional collaboration and cross-national knowledge exchange will be critical for synthesizing findings, reducing redundant or underpowered parallel efforts, enhancing the generalizability of the results, and informing practice and policy in diverse settings worldwide [41].

### 4.2. From Publication Outlets to Academic Influence

The present study further identified the most highly cited articles in the field (>300 citations, *n* = 11). These publications affirm the academic, clinical relevance, and value of Tai Chi by addressing major public health problems and issues involving falls in older adults [29,31], neurodegenerative diseases such as Parkinson’s disease [30], and chronic muscular pain syndromes such as fibromyalgia [37]. Most studies were either randomized controlled trials [29,30,31,36,37,38] or systematic reviews [33,34,35], and the majority appeared in leading medical journals, such as *The New England Journal of Medicine*, *Journal of the American Geriatrics Society*, and *Archives of Internal Medicine*. Their widespread citations demonstrate that Tai Chi, when linked to clinically meaningful outcomes, can achieve substantial visibility and influence in mainstream medicine.

In contrast, when the broader journal landscape is considered, Tai Chi studies remain heavily concentrated in journals classified within the Integrative and Complementary Medicine (CIM) category, notably *Evidence-Based Complementary and Alternative Medicine* and *Complementary Therapies in Medicine*. While this reliance on a single disciplinary platform has facilitated knowledge accumulation and exchange within a defined community, it also underscores the limited visibility of the field in top-tier medical outlets. Citation analysis further illustrates this complementary trend. Although CIM journals account for the majority of publications, interdisciplinary platforms often generate greater academic resonance. For instance, the *Journal of the American Geriatrics Society* ranked only 14th in publication volume but accrued the most citations, underscoring its practical relevance in gerontology. Similarly, outlets such as *Archives of Physical Medicine and Rehabilitation* and *Frontiers in Aging Neuroscience* report higher average citations per article than CIM-specific journals.

Mainstream sports and medical journals typically prioritize well-characterized interventions, including clearly specified dose and prescription parameters, well-defined comparator conditions, and outcome priorities aligned with their readership, such as mechanistically oriented or clinically salient endpoints. In contrast, Tai Chi studies often emphasize functional, symptom, or health-related quality-of-life outcomes, and prior reviews have identified recurrent challenges in trial design and reporting, including limited statistical power, ambiguously defined controls, short follow-up durations, and incomplete reporting of key intervention parameters [42,43,44]. Differences in conceptual framing, such as treating Tai Chi as exercise training versus mind–body therapy, may further shape outcome selection and cross-disciplinary visibility. Taken together, these patterns suggest that perceived journal fit, shaped by intervention characterization, reporting conventions, and outcome framing, may be a primary determinant of cross-disciplinary visibility and the broader impact of Tai Chi research beyond CIM venues.

### 4.3. Research Hotspots, Evolution and Future Perspectives

The top ten author keywords emphasize aging, functional and psychological outcomes, growing evidence-based approaches, and Tai Chi’s integration within mind–body exercise practices such as Qigong. Keyword co-occurrence analysis further demonstrated that Tai Chi research has evolved into a multi-dimensional framework. As shown in Figure 5, the analysis identified nine distinct clusters, which can be broadly summarized into five overarching themes: motor function, musculoskeletal conditions, psychological health, chronic diseases, and cognitive aging. These findings illustrate the expanding role of Tai Chi in both clinical and preventive health contexts and its growing recognition as a complementary therapy in exercise-based medicine.

Among the earliest themes to emerge, the green and red clusters highlighted Tai Chi’s functional value for older adults, focusing on balance, rehabilitation, falls, gait, and muscle strength. The prominence of kinematics and biomechanics positions this theme as one of the few areas in the network where mechanistic concepts are explicitly articulated, suggesting comparatively advanced methodological development. Building on this functional foundation, adjacent keywords signal the field’s gradual extension into exercise science and neuroscience, including applications in Parkinson’s disease, osteoporosis, and functional mobility. The purple cluster reflects the role of Tai Chi in chronic musculoskeletal pain conditions, such as rheumatoid arthritis, fibromyalgia, and osteoarthritis. These thematic focuses align with the most frequently cited studies in the field [29,30,36,37,38]. There is a growing consensus in the literature that Tai Chi is effective in reducing fall risk and improving balance [44]. Building on this evidence, an emerging body of work has examined style-related differences [45] and dose–response patterns [46] to optimize Tai Chi–based exercise prescriptions, indicating a shift toward greater methodological and clinical refinement within these themes.

The clusters on the left side of Figure 5 highlight Tai Chi’s engagement in chronic disease management. All four major types of chronic diseases identified by the World Health Organization (WHO), namely cardiovascular diseases, cancer, chronic respiratory diseases, and diabetes, were identified within the keyword network. In addition, the inclusion of COVID-19 among recent keywords reflects how Tai Chi emerged as a preferred home-based and highly accessible multicomponent rehabilitation option during the pandemic [47,48], alongside the renewed interest in telehealth-enabled rehabilitation [49]. Although these nodes have emerged more recently and the limited number and heterogeneity of existing studies still pose challenges for evidence synthesis [7], accumulating research suggests that Tai Chi has the potential to engage pathophysiological pathways relevant to chronic disease [50], while improving physical performance [7] and providing psychological benefits, including reductions in anxiety and depression [51], as well as enhanced quality of life [52]. Moreover, existing research indicates that these psychological benefits are not limited to clinical populations. Notably, the orange cluster, which focuses on mindfulness and meditation, includes the term college student, indicating that Tai Chi research has gradually expanded to younger age groups, particularly within the field of mental health [53,54].

In recent years, Tai Chi research has diversified, extending beyond traditional functional and psychological outcomes to new areas of inquiry, including cognitive health and the evolving context of global health. Studies have increasingly examined cognitive function, mild cognitive impairment, and dementia, reflecting the growing emphasis on brain health. This trend aligns closely with global demographic aging and the heightened awareness of cognitive decline as a pressing public health concern. Traditionally regarded as a mind–body exercise integrating elements of Chinese martial arts and meditation, Tai Chi emphasizes the coordination of movement, breathing, and mental focus. Its unique combination of aerobic activity, motor learning, and meditative engagement has been proposed as a pathway for enhancing cognitive function [55,56]. Emerging evidence suggests that Tai Chi may confer cognitive benefits to older adults [35,57,58,59].

The thematic structure revealed by the keyword analysis clarifies both the field’s development and the persistent gaps. Keyword patterns were dominated by clinical and symptom-oriented terms, whereas mechanistic inquiry, hard clinical endpoints (e.g., mortality and hospitalization), long-term trajectories, and population-level applications were underdeveloped. A decade ago, Harmer noted the fragmentation of the field and limited real-world progression [60]. Despite recent growth and diversification across populations, conditions, and prescriptions, structural gaps continue to constrain their translational potential. From a sports and exercise science perspective, these findings point to several underexploited directions: Tai Chi can be investigated as a movement-learning system, with coordination strategies, postural control, and movement variability as primary outcomes; growing interest in dose–response relationships and style-related variation also supports the development of exercise-prescription frameworks comparable to other training modalities (e.g., intensity, frequency, and adherence); and to strengthen real-world impact, future studies should pre-specify the theoretical framework (e.g., neuromuscular control or mind–body synergy) and align outcome selection and reporting accordingly, thereby improving interpretability across diverse populations and settings.

### 4.4. Limitations

Although our bibliometric analysis provides the most up-to-date and comprehensive perspectives on current research trends (Table 1), several limitations should be considered. First, reliance on a single bibliographic database (Scopus) may introduce selection bias. Although Scopus is a comprehensive and widely used index, its coverage is dominated by internationally oriented biomedical journals and only partially extends to regional or locally indexed outlets (e.g., Chinese-language journals), potentially underrepresenting culturally embedded or region-specific aspects of Tai Chi research. Second, to maintain a focused scope, the search was restricted to article titles, which increased specificity for Tai Chi–focused studies but may have omitted interventions embedded within broader physical activity, rehabilitation, or health-promotion frameworks that did not explicitly mention Tai Chi in the title. Third, the analysis depended on author-defined keywords and citation-based indicators. Despite unification efforts, inconsistencies in terminology remained, as some terms appeared at different levels of specificity (e.g., “cognition,” “cognitive function,” “executive function”). Such variability may distort clustering results by inflating broad categories while obscuring specialized but emerging topics. In addition, citation-based metrics are susceptible to citation bias, including the disproportionate influence of highly cited review articles and clinically popular topics, as well as author and journal self-citations. The coexistence of primary studies and review articles inevitably introduces some overlap because reviews aggregate underlying trials and can amplify the apparent prominence of certain themes.

## 5. Conclusions

Drawing on 2253 publications over nearly five decades, this study provides an updated overview of Tai Chi research. Output has increased steadily since the late 1970s, with growth largely concentrated since 2013, mirroring trends in other mind–body therapies. Globally, Tai Chi research is led by China and the United States and is largely driven by a small number of clusters concentrated in specific regions. In keyword analysis, nine software-generated keywords clusters were manually synthesized into five core themes: motor function, musculoskeletal conditions, psychological health, chronic diseases, and cognitive aging, reflecting the multidimensional role of Tai Chi in health promotion. The increased visibility of trial- and review-related terms points to growing attention to study design and evidence synthesis, alongside an expanding theme scope. Nevertheless, stronger international collaboration and methodological refinement, including more mechanism-informed study designs, longer-term follow-up, and greater emphasis on population- and implementation-level research, are crucial for realizing the full potential of Tai Chi for global health.

## Figures and Tables

**Table 1:** Summary of previous and present bibliometric studies on Tai Chi.

Studies (Year) (*n*)	Search Terms	Database	Study Period, Language Limit
Yang et al. (2015) [18] (***n*** = 507)	Tai Chi, Taiji, Tai Ji, Tai-ji, Tai Chi Chuan, Tai Chi Quan, Taijiquan	PubMed, the Cochrane Library, CNKI, VIP, Sino-Med, Wanfang	1958 to 2013, no language limit
Yang et al. (2021) [19] (***n*** = 1018)	Tai Chi, Taiji, Tai Ji, Tai-ji, Tai Chi Chuan, Tai Chi Quan, Taijiquan	PubMed, Cochrane Library, EMBASE, Medline, Web of Science, CNKI, VIP, Sino-Med, Wanfang	2010 to 2020, no language limit
You et al. (2021) [20] (***n*** = 1078)	Tai Chi, Tai-Ji, Taiji, Tai Ji Quan, Chi, Tai, Taichi, Taijiquan, Tai Chi Chuan, Ji Quan, Tai, Quan, Tai Ji, Tai-Yi	Web of Science	1980 to 2020, no language limit
Wang et al. (2022) [21] (***n*** = 1936)	Tai Chi, Taichi, Tai-Chi, Tai Ji, Taiji, Tai-Ji, Taijiquan, Taichiquan, Taijichuan, Taichichuan”	Web of Science	1991 to 2021, English only
Li et al. (2025) [22] (***n*** = 941)	Tai Chi, Taijiquan, Tai Chi Quan	Web of Science	2004 to 2024, English only
Huang et al. (2025) (current study) (***n*** = 2253)	Tai Chi, Taichi, Tai-Chi, Tai Ji, Taiji, Tai-Ji, T’ai-Chi, Taichiquan, Taijiquan, Taichichuan, Taijichuan	Scopus	1978 to 2025, no language limit

Note: ***n***, the number of included studies, CNKI, China National Knowledge Infrastructure; VIP, Chinese Scientific Journal Database.

**Table 2:** Top 15 countries by publication output in Tai Chi research.

Rank	Country	Publications	Citations		Rank	Country	Publications	Citations
1	China	892 (40%)	15,841		9	Germany	45 (2%)	1122
2	United States	687 (30%)	29,485		10	Japan	43 (2%)	733
3	Hong Kong	179 (8%)	7764		11	Spain	27 (1%)	357
4	Taiwan	155 (7%)	5335		12	Brazil	26 (1%)	366
5	Australia	116 (5%)	3955		13	Malaysia	26 (1%)	238
6	United Kingdom	113 (5%)	3039		14	Thailand	26 (1%)	558
7	South Korea	90 (4%)	3030		15	Italy	24 (1%)	189
8	Canada	89 (4%)	2831					

**Table 3:** Most-cited documents on Tai Chi (1978–2025).

Author (year)	Citations	Title	Journal (IF, JCR Quartile)
Wolf (1996) [29]	963	Reducing frailty and falls in older persons: An investigation of Tai Chi and computerized balance training	*Journal of the American Geriatrics Society* (IF: 4.5, Q1)
Li (2012) [30]	676	Tai Chi and postural stability in patients with Parkinson’s disease	*The New England Journal of Medicine* (IF: 78.5, Q1)
Li (2005) [31]	554	Tai Chi and fall reductions in older adults: A randomized controlled trial	*The Journals of Gerontology. Series A, Biological Sciences and Medical Sciences* (IF: 3.8, Q1)
Jahnke (2010) [32]	445	A comprehensive review of health benefits of qigong and Tai Chi	*American Journal of Health Promotion* (IF: 2.4, Q2)
Wolfson (1996) [36]	432	Balance and strength training in older adults: Intervention gains and Tai Chi maintenance	*Journal of the American Geriatrics Society* (IF: 4.5, Q1)
Wang (2004) [33]	413	The Effect of Tai Chi on Health Outcomes in Patients with Chronic Conditions: A Systematic Review	*Archives of Internal Medicine* (IF: 17.3, 2014)
Wang (2010) [37]	370	A randomized trial of Tai Chi for fibromyalgia	*The New England Journal of Medicine* (IF: 78.5, Q1)
Wang (2010) [34]	344	Tai Chi on psychological well-being: Systematic review and meta-analysis	*BMC Complementary and Alternative Medicine* * (IF: 3.4, Q1)
Wayne (2014) [35]	329	Effect of Tai Chi on cognitive performance in older adults: Systematic review and meta-analysis	*Journal of the American Geriatrics Society* (IF:4.5, Q1)
Wolf (1997) [38]	314	The effect of Tai Chi Quan and computerized balance training on postural stability in older subjects	*Physical Therapy* (IF = 3.3, Q1)
Jin (1992) [39]	313	Efficacy of Tai Chi, brisk walking, meditation, and reading in reducing mental and emotional stress	*Journal of Psychosomatic Research* (IF = 3.3, Q2)

Note: Only documents with at least 300 citations are shown (*n* = 11); IF, Impact Factor from Journal Citation Reports 2024. * *BMC Complementary and Alternative Medicine* was renamed *BMC Complementary Medicine and Therapies* in 2020.

**Table 4:** Top 15 journals by the number of publications.

Rank	Journal	Region	Pub.	Citations (Citations Per Paper)	IF (2024)	JCR Categories	Quartile
1	*Evidence-Based Complementary and* *Alternative Medicine*	England	64	1587 (25)	2.7 (2021)	Integrative and Complementary Medicine	Q3
2	Journal of Alternative and Complementary Medicine *	USA	50 + 8 *	1862 + 24 * (33, Top 4)	1.7	Integrative and Complementary Medicine	Q3
3	*Complementary Therapies in Medicine*	England	42	1205 (28)	3.5	Integrative and Complementary Medicine	Q1
4	*Medicine*	USA	40	294 (7)	1.4	Medicine, General and Internal	Q2
5	*International Journal of Environmental* *Research and Public Health*	Switzerland	34	599 (18)	4.6 (2021)	Public, Environmental and Occupational Health	Q1
6	*Archives of Physical Medicine and* *Rehabilitation*	USA	31	2524 (81, Top 2)	3.7	Rehabilitation Sport Sciences	Q1
7	*PLoS ONE*	USA	30	1091 (36, Top 3)	2.6	Multidisciplinary Sciences	Q2
8	*Frontiers in Aging Neuroscience*	Switzerland	28	662 (24)	4.5	Neurosciences Geriatrics and Gerontology	Q1
9	*Chinese journal of rehabilitation medicine*	China	27	108 (4)	-	-	-
10	*BMJ Open*	England	26	315 (12)	2.3	Medicine, General and Internal	Q2
11	*Complementary Therapies in Clinical Practice*	Netherlands	26	782 (30)	3	Integrative and Complementary Medicine	Q2
12	*Journal of Aging and Physical Activity*	USA	25	849 (34, Top 5)	1.5	Gerontology Sport Sciences Geriatrics and Gerontology	Q3
13	*Research in Sports Medicine*	England	25	541 (22)	1.9	Sport Sciences	Q2
14	*Journal of the American Geriatrics Society*	USA	24	4473 (186, Top 1)	4.5	Gerontology Geriatrics and Gerontology	Q1
15	*Trials*	England	24	241 (10)	2	Medicine, Research and Experimental	Q3

Note: Pub, Publications, IF, Impact factor values were retrieved from 2024 Journal Citation Reports. The most recently reported JCR Impact Factor is listed for journals that have discontinued JCR. * renamed *Journal of Integrative and Complementary Medicine* in 2022.

## Data Availability

The original contributions presented in this study are included in the article. Further inquiries can be directed to the corresponding author.
